
JOURNAL INFORMATION
==============================
NLM Title Abbreviation: Innov Surg Sci
Journal ID: Innov Surg Sci
NLM Journal ID: 101708165
Journal ID: iss

Innovative Surgical Sciences

EISSN: 2364-7485
Publisher: De Gruyter

ARTICLE INFORMATION
==============================
PMCID: PMC13068487
DOI: 10.1515/iss-2026-2006
Article ID: iss-2026-2006
Article version: 1
Subjects: Article

6. Neurosurgery

Electronic publication date: 2026 Apr 10
Publication date: 2026 Apr
Volume: 11
Issue: Suppl1
First page: 396
Last page: 410
Copyright: © 2026 by Walter de Gruyter Berlin/Boston
Copyright year: 2026
Copyright holder: De Gruyter
License: This work is licensed under the Creative Commons Attribution 4.0 International License.
License URL: https://creativecommons.org/licenses/by/4.0/

Page count: 15

==============================

DE GRUYTER BRILL

**Stimulated Raman Histology for intraoperative detection of meningioma infiltration in dural margins**
ID: E-DCK-203

**Neurochirurgie / Neurosurgery**
**KI/Robotik/Navigation in der Neurochirurgie / AI / Robotics / Navigation in Neurosurgery**

Claire Wang[1], David Reinecke[1], Abdulkader Al-Shughri[2], Nina Müller[1], Anna-Katharina Meißner[1], Gina Fürtjes[1], Lili Leyer[1], Zachari Gueorguiev[1], Jonathan Kottlors[3], Roland Goldbrunner[1], Felix Sahm[4], Volker Neuschmelting[1]

[1]Department of General Neurosurgery, Center for Neurosurgery, University Hospital Cologne, Cologne, Deutschland, [2]Department of Pathology, Institute for Neuropathology, University Hospital Cologne, Cologne, Deutschland, [3]Department of Neuroradiology, Institute for Diagnostic and Interventional Radiology, University Hospital Cologne, Cologne, Deutschland, [4]Department of Neuropathology, Institute for Pathology, University Hospital Heidelberg, Heidelberg, Deutschland

**Background:**
Meningiomas are the most common intracranial tumors, and while gross total resection (GTR) of meningiomas including their dural attachments is the primary treatment of choice, recurrence rates are highly variable and remain a persistent clinical challenge. The grading of extent of resection (EOR) in meningioma surgery currently relies on the surgeon’s macroscopic assessment utilizing tools like white light microscopy, MRI-based neuronavigation, ultrasound or fluorescence-guided surgery, all of which fail to reliably detect microscopic tumor cell remnants beyond visible tumor margins. Given that recurrences presumably arise from such residual microscopic tumor infiltration within the primary arachnoid resection bed and the dural infiltration zone (dural tail), there is need for more precise methods of microscopic resection control. This study aims to evaluate the feasibility of emerging Stimulated Raman Histology (SRH) for rapid intraoperative visualization of residual tumor and characterization of dural infiltration patterns.

**Material and methods:**
In this monocentric prospective observational study, patients with primary or recurring convexity-associated meningiomas with a neurooncological indication for complete resection were included.
For SRH image generation, up to 4 tissue samples (<3mm$^3$) were obtained from the dural resection margins, which had been assessed as tumor-free by the surgeon, as well as samples from the tumor core serving as tumor reference. These tissue specimens were then imaged with a portable stimulated raman scattering microscope. Additionally, preoperative MRI images were integrated into the neuronavigation system to enable spatial localization of the resection margin samples. Subsequent histopathological processing with conventional H&E staining of the samples served as the ground truth for later assessment of the diagnostic accuracy of SRH imaging and AI-based analysis algorithms.

**Results:**
Currently, 36 patients have been enrolled, including 18 conventionally diagnosed with CNS WHO grade 1 meningiomas, 17 with CNS WHO grade 2 and 1 with CNS WHO grade 3. Of these, 29 were female and 7 male. 9 of the 36 patients (25%) intended for complete resection showed microscopic tumor infiltration in

Open Access. © 2026 The Author(s), published by De Gruyter. This work is licensed under the Creative Commons Attribution 4.0 International License.

at least one resection margin upon histopathological processing. In total, we generated 220 SRH images, 131 of which originated from resection margins assessed as tumor-free. Of the 121 margin samples processed histopathologically, 12 (9.9%) still contained residual tumor cells. The MRI correlation analyses are still ongoing.

**Conclusion:**

Despite Simpson Grade I resection, focal microscopic tumor remnants were detectable in the resection bed of numerous patients, highlighting the limitations of current resection control methods and the need for precise dural margin assessment. SRH enables intraoperative imaging of critical resection margins on the microscopic level, thus allowing rapid visualization of tumor remnants not identifiable macroscopically. Further work is required to optimize tissue sampling strategies and advance the development of a user-independent AI-based analysis algorithm for rapid identification of tumor-infiltrated dural margins and enable surgical guidance.

DE GRUYTER BRILL

**GPT in OPS-Coding for lumbar Sequestrectomy: Can the AI keep up?**
ID: E-DCK-457

**Neurochirurgie / Neurosurgery**
**KI/Robotik/Navigation in der Neurochirurgie / AI / Robotics / Navigation in Neurosurgery**

Sebastian Lehmann[1], Florian Wilhelmy[1], Nikolaus von Dercks[2], Erdem Güresir[1], Johannes Wach[1]

[1]Department of Neurosurgery, University Hospital Leipzig, Leipzig, Deutschland, [2]Medical Management, University Hospital Leipzig, Leipzig, Deutschland

**Background:**
Operation Procedure Classification System (OPS) coding is the basis for the remuneration of surgical disciplines in the German diagnosis-related groups (DRG)-based healthcare system. In this study, we analyse the ability of the large language model (LLM) GPT to sufficiently derive OPS coding from the operative report (OR).

**Material and methods:**
We retrospectively investigated the OR of 100 patients who underwent lumbar microdiscectomy. The codes encrypted by the surgeons postoperatively, as well as the coding by our professional coders were extracted. Also GPT-4o, GPT CodeMedic and GPT-5 were given the task to determine the correct OPS coding based on the OR. The results were evaluated by sufficient coding basic coding and optimal coding (comprehensive coding). The results of the four groups then were compared using cross tabulation. Secondary, miscoding and its causes were analyzed.

**Results:**
In our analysis, the certified coders tended to be superior to the LLMs in coding lumbar sequestrectomies for sufficient coding (GPT-4o $p$=0.06; GPT CodeMedic $p$=0.09), GPT-5 $p$=0.25) and showed significant superiority in optimal coding for GPT 4-Versions (GPT-4o$p$=0.06; GPT CodeMedic $p$<0.01), GPT-5 $p$=0.06. No statistically significant differences were found in the results for optimal coding between surgeons and the LLMs, or between the LLMs themselves. Advanced GPT Versions showed fewer hallucinations (GPT-4o = 18%; GPT CodeMedic = 15%, GPT-5 = 8%).

**Conclusion:**
GPT is capable of extracting a high rate of correct OPS codes from an OR. While certified coders are still unreached in the rate of both sufficient and optimal coding, the LLMs perform equally to the surgeons regarding optimal coding.

DE GRUYTER BRILL

**Shape Radiomics and their Relation to Outcome in Spontaneous Intraventricular Hemorrhage**
ID: E-DCK-491

**Neurochirurgie / Neurosurgery**
**KI/Robotik/Navigation in der Neurochirurgie / AI / Robotics / Navigation in Neurosurgery**

Tim Wende[1], Gordian Prasse[2], Johannes Wach[1], Erdem Güresir[1], Alim Basaran[1], Martin Vychopen[1]

[1]Klinik für Neurochirurgie, Universitaetsklinikum Leipzig, Leipzig, Deutschland, [2]Institut für Neuroradiologie, Universitaetsklinikum Leipzig, Leipzig, Deutschland

**Background:**
Spontaneous intraventricular hemorrhage (sIVH) without underlying intracerebral hemorrhage is a very rare condition, associated with a high in-hospital mortality rate of approximately 40%. We aimed to examine the shape radiomic features of sIVH and their correlation with neurological outcome.

**Material and methods:**
We retrospectively reviewed all patients with spontaneous intracranial hemorrhage treated within our institution between 2018 and 2023. Patients with intracerebral hematoma or hemorrhage due to an underlying pathology, such as tumor or artveriovenous malformation, were excluded. A total of 34 patients was eligible for analysis. Outcome was assessed using the modified Rankin Scale (mRS) and dichotomized for favourable (mRS ≤ 3) and unfavourable outcome (mRS > 3).

**Results:**
Favorable outcome at discharge was achieved in only 6/34 patients (17%). In-hospital mortality was 35%. In multivariate binary regression analysis, only hematoma surface area correlated with in-hospital mortality ($p = 0.021$). The cutoff was calculated at 144.54 $cm^2$ (AUC 0.773, OR 9.000, CI 1.787–45.336, $p = 0.008$). There was no independent correlation for any variable with favourable outcome at discharge.

**Conclusion:**
Spontaneous intraventricular hemorrhage remains a devastating disease. Beyond established prognostic factors, hematoma surface area, but not volume, may be a suitable candidate for outcome prognostication.

DE GRUYTER BRILL

**Validity of Recommendations by a LLM for Interdisciplinary Board Decisions in Neurooncology.**
ID: E-DCK-83

**Neurochirurgie / Neurosurgery**
**KI/Robotik/Navigation in der Neurochirurgie / AI / Robotics / Navigation in Neurosurgery**

Maria Goldberg[1], Viktor Eisenkolb[1], Amir Kaywan Aftahy[2], Chiara Negwer[1], Hanno S. Meyer[3], Jens Gempt[3], Bernhard Meyer[1], Arthur Wagner[1]

[1]Department of Neurosurgery, School of Medicine, Klinikum rechts der Isar, Technical University Munich, Munich, Deutschland, [2]International Neuroscience Institute, Hannover, Deutschland, [3]Department of Neurosurgery, University Medical Center Hamburg-Eppendorf, Hamburg, Deutschland

**Background:**
This study aims to evaluate the stylistic and structural equivalence of Artificial Intelligence (AI)-generated summaries, particularly those by Large Language Models (LLMs) like ChatGPT, compared to traditional human-generated case summaries in neuro-oncological board decisions. The primary goal is to explore the stylistic alignment between AI-generated and human-authored summaries from board meeting audio recordings.

**Material and methods:**
The study compares 30 traditional human-generated case summaries with 30 AI-generated summaries based on board meeting audio recordings. Two expert raters, blinded to the source of the summaries, evaluated a total of 60 cases. A Likert scale was used to assess the plausibility, linguistic style, evidence adherence, and reference accuracy of the summaries.

**Results:**
The results indicated that both LLM-generated and human-reviewed summaries demonstrated consistently high performance across all criteria evaluated. The general plausibility ratings were comparable (LLM: 4.7, Human: 4.73, $p = 0.959$). Linguistic style ratings also showed similarity (LLM: 4.87, Human: 4.97, $p = 0.512$). In terms of adherence to evidence, the means were close (LLM: 4.8, Human: 4.87, $p = 0.541$). Reference accuracy was slightly higher for AI-generated summaries (LLM: 4.97, Human: 4.9, $p = 0.664$). These findings were consistent with the results from Rater 2, and statistical analysis using Kendall's tau showed no significant differences between methods ($p > 0.05$).

**Conclusion:**
The study finds that LLM-generated summaries can effectively emulate the style and structure of human-authored ones, indicating their promise as an additional tool in neuro-oncology. These AI models can enhance documentation quality and serve as valuable support in clinical settings. While further research is necessary to explore broader applications, LLMs offer exciting potential as a complement to traditional decision-making processes.

DE GRUYTER BRILL

### In-Vivo Hyperspectral Imaging of Edema and Cortical Oxygenation in Meningiomas: Interim Analysis

ID: E-DCK-300

**Neurochirurgie / Neurosurgery**
**Minimalinvasive Techniken / Minimally Invasive Techniques**

Johannes Wach[1], Martin Vychopen[1], Anne-Katrin Pfahl[2], Hannes Köhler[2], Andreas Melzer[2], Ferdinand Weber[2], Erdem Güresir[2]

[1]Klinik und Poliklinik für Neurochirurgie, Universitätsklinikum Leipzig, Leipzig, Deutschland, [2]ICCAS Leipzig, Leipzig, Deutschland

**Background:**
Peritumoral brain edema (PBE) is common in intracranial meningiomas (IM) and has been linked to postoperative morbidity, yet its impact on cortical oxygenation remains unclear. We investigated the association between PBE and local tissue oxygenation using intraoperative in-vivo hyperspectral imaging (HSI).

**Material and methods:**
Twenty-nine patients undergoing microsurgical IM resection with intraoperative HSI were prospectively analyzed. Peritumoral cortical oxygen saturation ($StO_2$) was quantified and correlated with preoperative MRI parameters, including edema volume and contrast-enhancement intensity. Patients were stratified by PBE presence and histopathological subtype (meningotheliomatous vs. non-meningotheliomatous). Group comparisons used t-tests; correlations employed Pearson coefficients.

**Results:**
PBE was present in 17 (58.6%) cases. Patients with PBE exhibited significantly lower cortical oxygenation than those without (mean $StO_2$ 0.49 ± 0.13 vs. 0.59 ± 0.10, $p = 0.048$). Within the PBE group, edema volume correlated positively with $StO_2$ ($r = 0.58$, $p = 0.015$), whereas tumor contrast-enhancement intensity correlated inversely with $StO_2$ ($r = 0.59$, $p = 0.013$). No significant correlation was observed in patients without PBE ($r = –0.26$, $p = 0.18$). Meningotheliomatous IMs showed a trend toward lower $StO_2$ compared with non-meningotheliomatous tumors (0.51 ± 0.17 vs. 0.55 ± 0.07, $p = 0.05$).

**Conclusion:**
PBE in IMs is associated with impaired cortical oxygenation. Larger PBEs paradoxically showed higher $StO_2$, suggesting compensatory mechanisms, while strong tumor enhancement correlated with local hypoxia. These findings suggest that intraoperative HSI may serve as a promising tool for characterizing tumor–brain interactions and support further investigation into PBE pathophysiology.

DE GRUYTER BRILL

**Chronic implantation of microelectrode arrays as a brain-computer-interface: a technical note.**
ID: E-DCK-165

**Neurochirurgie / Neurosurgery**
**Neuroprothetik / Neuroprosthetics**

Arthur Wagner[1], Viktor Eisenkolb[1], Simon Jacob[2], Bernhard Meyer[1]

[1]Department of Neurosurgery, University Hospital, Technical University Munich School of Medicine, Munich, Deutschland, [2]Translational Neurotechnology Laboratory, Department of Neurosurgery, University Hospital, Technical University Munich School of Medicine, Munich, Deutschland

**Background:**
The chronic implantation of brain–computer interfaces (BCIs) facilitates stable, high-fidelity neuronal recordings over extended periods of time. Planar microelectrode arrays (MEAs), or “Utah arrays,” are commonly used for intracortical signal acquisition. Implantation of multiple arrays across distinct cortical targets within one hemisphere presents specific technical and clinical challenges. Here, we describe the surgical workflow for chronic implantation of multiple Utah arrays in two patients and report safety and signal-quality outcomes.

**Material and methods:**
Two patients (MB and MM) underwent chronic MEA implantation within a translational research program at TUM University Hospital after ethical approval and written informed consent in 2022 and 2025. Preoperative planning included structural magnetic resonance imaging and navigated transcranial magnetic stimulation (nTMS) mapping for localization of functional targets. Both patients received four individual MEAs with 64 microelectrodes each, totalling 256 intracortical electrodes per patient. MB (female) had presented with aphasia after having suffered a left hemisphere media territory stroke 6 years prior to implantation and received 4 MEAs in the middle frontal, inferior frontal, supramarginal, and angular gyri. MM (male) presented with tetraparesis after suffering a high level spinal cord injury in a motor vehicle accident 9 years prior to implantation and received 4 MEAs in the dorsal premotor cortex, hand knob (2x), and posterior parietal cortex.

**Results:**
The duration of the chronic implantation has currently amounted to 39 months for MB and 2 months for MM. Intraoperatively, frameless neuronavigation allowed for localization of the aforementioned prespecified cortical targets. Via a myocutaneous flap, tailored craniotomy and durotomy, MEAs were inserted with a pneumatic pulse under direct microscopic visualization, establishing the MEA-cortex interface for electrophysiological data acquisition. Wires were routed along the craniotomy margins tension-free and secured to minimize mechanical strain on the MEAs. The pedestal was screwed to adjacent cortical bone after drilling the external table for an optimized profile and stability. A stab incision permitted percutaneous pedestal externalization, establishing the interface for the headstage. MM reported paresthesia and experienced speech arrests on the first postoperative day, which fully resolved with antiepileptic drug treatment within 15 days. He was discharged on postoperative day 9 after an otherwise uneventful course. MB was discharged on postoperative day 3 after an uneventful course. After 15 months, MB suffered from wound breakdown around the pedestal, necessitating surgical wound debridement under local anesthesia and intravenous antibiotic treatment. With meticulous wound management including regular cleaning and antiseptic dressing, no further tissue breakdown was recorded. No further complications from MEA implantation were recorded otherwise. Optimal signal quality over time was recorded in MB in 3 out of 4 MEAs, and in MM in all MEAs.

**Conclusion:**
Chronic implantation of MEAs across distinct cortical areas is safe. A standardized workflow—combining imaging-based functional nTMS mapping, intraoperative neuronavigation, and structured postoperative surveillance—supports reliable, long-term intracortical signal acquisition. These technical case reports provide practical guidance for chronic implantation strategies and postoperative care for invasive BCIs to support continued clinical translation.

DE GRUYTER BRILL

**Cervical Spinal Cord Stimulation following Spinal Cord Injury: A Systematic Review**
ID: E-DCK-492

**Neurochirurgie / Neurosurgery**
**Neuroprothetik / Neuroprosthetics**

Maximilian Wankner[1], Petra Heiden[1], Pablo Andrade[1]

[1]Uniklinik Köln, Köln, Deutschland

**Background:**
Cervical spinal cord injury (SCI) results in profound motor and autonomic impairment, with limited natural recovery even under intensive rehabilitation. While epidural and transcutaneous spinal cord stimulation have demonstrated functional benefits in thoracic and lumbosacral injuries, their application to the cervical cord is more recent. Given the high clinical relevance of upper limb and respiratory function, a systematic review of the evidence is needed.

**Material and methods:**
We conducted a systematic review registered in PROSPERO (ID: CRD420251088804) in accordance with PRISMA guidelines. PubMed, Embase, and Web of Science were searched for preclinical and clinical studies investigating cervical spinal cord stimulation (epidural, intraspinal, or transcutaneous) in SCI. Only studies reporting functional outcomes were included. Data were extracted on study and patient characteristics, stimulation protocols, outcome measures, adverse effects, and methodological quality. Risk of bias was assessed using RoB 2 for randomized controlled trials and ROBINS-I for non-randomized studies.

**Results:**
31 studies published between 2013 and 2025 were included, comprising 119 animal and 156 human subjects. Interventions varied across invasive (epidural, intraspinal) and non-invasive (transcutaneous) modalities, with electrode placements most commonly targeting the C3–C7 segments. Functional outcomes were heterogeneous, but improvements were most frequently reported in hand and arm motor scores. Some studies also described sensory gains, autonomic improvements, and, in single cases, respiratory benefits. Adjunctive interventions including brain–computer interface, motor imagery, cortical stimulation, and robotic exoskeleton training were often combined with stimulation. Most studies reported only minor adverse events. Risk of bias was generally serious, with limitations including small sample sizes, heterogeneous cohorts, lack of blinding, and absence of sham controls.

**Conclusion:**
Current evidence suggests that cervical spinal cord stimulation can enhance motor function in patients with cervical SCI through assistive and therapeutic effects, with preliminary signals of benefit across sensory and autonomic domains. However, the certainty of evidence remains low due to methodological limitations. Larger sham-controlled trials are needed to establish efficacy, clarify mechanisms, optimize stimulation protocols, and determine which patient populations are most likely to benefit. Integration with rehabilitation strategies and assessment of broader outcomes including independence, quality of life, and cost-effectiveness will be critical for translation into clinical practice.

DE GRUYTER BRILL

**AI-guided Stimulated Raman Histology for Rapid Margin Control during Brain Metastases Surgery**
ID: E-DCK-208

**Neurochirurgie / Neurosurgery**
**ZNS Metastasen / CNS Metastases**

Lili Leyer[1], Lisa Irina Körner[2], Anna-Katharina Meißner[1], Nina Müller[1], Thomas Roetzer-Pejrimovsky[3], Abdulkader Al Shughri[4], Thomas Stehle[4], Gina Fürtjes[1], David Reinecke[1 5], Barbara Kiesel[2], Claire Wang[1], Hannah Neuschmelting[6], Ammar Mallouhi[7], Reinhard Büttner[8], Karl Rössler[2], Roland Goldbrunner[1], Georg Widhalm[2], Volker Neuschmelting[1]

[1]University Hospital Cologne, Center for Neurosurgery, Cologne, Deutschland, [2]Medical University Vienna, Department of Neurosurgery, Vienna, Österreich, [3]Medical University Vienna, Department of Neurology, Division of Neuropathology and Neurochemistry, Vienna, Österreich, [4]University Hospital Cologne, Department of Pathology, Institute for Neuropathology, Cologne, Deutschland, [5]New York University Grossman School of Medicine, Department of Neurosurgery, New York, Vereinigte Staaten, [6]University Hospital Cologne, Department of Neuroradiology, Institute for Diagnostic and Interventional Radiology, Cologne, Deutschland, [7]Medical University Vienna, Department of Neuroradiology and Musculosceletal Radiology, Vienna, Österreich, [8]University Hospital Cologne, Department of Pathology, Cologne, Deutschland

**Background:**
The extent of resection (EOR) impacts local control in brain metastasis (BM) surgery. The surgeons' subjective intraoperative margin assessment often causes incomplete resections[1]. This study evaluates stimulated Raman histology (SRH) with artificial intelligence (AI) for rapid intraoperative resection margin assessment in BM surgery.

**Material and methods:**
In this international, bicentric prospective observational diagnostic study, up to six 1mm3 tissue specimens from the resection cavity were sampled by the respective surgeon macroscopically proposed as tumor-free in adult patients undergoing non-eloquent BM resection. An additional tumor core specimen served as tumor reference (positive control). The SRH images of the margin samples were classified as 'tumor,' 'non-tumor,' or 'low quality' by a previously validated brain tumor-trained AI algorithm[2]. Conventional histopathologyserved as the reference standard; EOR was assessed by postoperative MRI (< 72 h).

**Results:**
We enrolled 75 patients with 330 margin samples (28.0% posterior fossa, 14.7% recurrencies), residual tumor was found in 18.7% of patients. Separate thresholds improved AI performance: supratentorial (44) vs. infratentorial (66) reached a balanced accuracy of 94.2% vs. 87.0% (sensitivity 93.6% vs. 80.7%, specificity 94.8% vs. 93.2%, area under the curve 93.8% vs. 86.9%, negative predictive value 97.4% vs. 88.2%). False positives were significantly more frequent in samples from the posterior fossa (p=.001). The surgeons' assessment balanced accuracy was 50.0%, postoperative MRI 55.0% and found inferior to SRH plus AI.

**Conclusion:**
AI-guided SRH imaging enables rapid intraoperative margin assessment outperforming both the surgeons' and MRI evaluation. A higher threshold is needed for metastases in the posterior fossa. Negative AI analysis results reliably indicate tumor-free tissue.

[1]Kiesel B et al., *World Neurosurg.* 2020 Nov;143:e365-e373.
[2]Reinecke D et al., *Acta Neuropathol Commun* 2022;10:109.

DE GRUYTER BRILL

**Applying pathology foundation models on Stimulated Raman Histology for diagnosis of brain metastases**
ID: E-DCK-348

**Neurochirurgie / Neurosurgery**
**ZNS Metastasen / CNS Metastases**

Anna-Katharina Meißner[1], David Reinecke[1], Jakob Strähle[2], Tim Lenz[3], Arianna Bonetti[3], Vidhya Sainath[3], Cheng Jiang[4], Asadur Chowdury[4], Gina Fürtjes[1], Nina Müller[1], Lili Leyer[1], Abdulkadher Al Shughri[5], Zunamys Carrero[3], Jürgen Beck[2], Roland Goldbrunner[1], Todd Hollon[4], Jakob Nikolas Kather[3 6 7 8], Volker Neuschmelting[1]

[1]Dept. of General Neurosurgery, Center for Neurosurgery, Faculty of Medicine and University Hospital Cologne, University of Cologne, Cologne, Germany, Cologne, Deutschland, [2]Department of Neurosurgery, Medical Center, University of Freiburg, 79106, Freiburg, Germany, Freiburg, Deutschland, [3]Else Kroener Fresenius Center for Digital Health, Faculty of Medicine and University Hospital Carl Gustav Carus, TUD Dresden University of Technology, 01307 Dresden, Germany, Dresden, Deutschland, [4]Machine Learning in Neurosurgery Laboratory, Department of Neurosurgery, University of Michigan, Ann Arbor, MI, USA, Computational Medicine and Bioinformatics, University of Michigan, Ann Arbor, MI, USA, Ann Arbor, Vereinigte Staaten, [5]Institute for Neuropathology, University Hospital Cologne, Cologne, Germany, Cologne, Deutschland, [6]Medical Oncology, National Center for Tumor Diseases (NCT), University Hospital Heidelberg, Heidelberg, Germany, Heidelberg, Deutschland, [7]Pathology & Data Analytics, Leeds Institute of Medical Research at St James's, University of Leeds, Leeds, United Kingdom, Leeds, Großbritannien, [8]Department of Medicine II, Faculty of Medicine and University Hospital Carl Gustav Carus, TUD Dresden University of Technology, 01307 Dresden, Germany, Dresden, Deutschland

**Background:**
Intraoperative diagnosis of brain metastases, ideally including subtype and molecular status, is crucial for guiding surgical decision-making and enabling personalized neurosurgical strategies. Conventional rapid frozen sections are limited by turnourand time, reproducibility and are currently not feasible for molecular diagnostics. Stimulated Raman Histology (SRH) is a label-free optical imaging technique suitable for intraoperative use and has shown potential for tumor classification using artificial Intelligence (AI). However, the subtyping of brain metastases is not feasible yet with the estalished AI approaches.
This study evaluates the performance of pathology foundation models—pretrained on hematoxylin and eosin (H&E) stained whole-slide images—when applied to SRH data for intraoperative classification of brain metastases.

**Material and methods:**
We designed a two-level diagnostic pipeline consisting of patch-level feature extraction and patient-level prediction. Feature extraction was performed using several state-of-the-art pathology foundation models, pretrained on large scale H&E histopathology data. Extracted features were aggregated into patient-level predictions through different pooling strategies and ensemble approaches. Model development and internal validation were performed using SRH data from two German neurosurgical centers. Five-fold cross-validation was applied to assess internal robustness. External validation was carried out on an independent cohort. Performance was quantified by area under the receiver operating characteristic curve (AUROC).

**Results:**
A total of 275 patients with 1407 SRH images from to German neurosurgical centers were used for model development and internal validation. The external validation cohort comprised 151 patients with 826 images from a US neurosurgical department. We analzyed brain metastases on two classification levels: (1) main histomorphological classes - including adenocarcinoma, melanoma, sarcoma - and (2) primary tumors - further stratifying metastases according to their site of origin. The best performing models using UNI, UNI2[1]and VIRCHOW2[2] as feature extarctors achieved an AUROC of 0.67-0.85 for histomorphological classification and 0.64-0.83 for primary tumor prediction. Ensemble approaches combining multiple foundation models further improved the predictive performance (AUROC up to 0.89).

**Conclusion:**
This work demonstrates, for the first time, that foundation models pretrained on H&E histology can be successfully transferred to SRH for intraoperative diagnosis of brain metastases. Our findings indicate that these models achieve robust classification performance and generalize across independent patient cohorts, underscoring their translational potential for real-time clinical workflows for personalized surgical approaches. For complex down-stream tasks as the subclassification of adenocarcinomas, where predictive performance was lower, further validation with large multicenter datasets will be essential.

DE GRUYTER BRILL

**Fibroblast growth factors are increased in BRAF mutated melanoma brain metastases**
ID: E-DCK-487

**Neurochirurgie / Neurosurgery**
**ZNS Metastasen / CNS Metastases**

Marco Timmer[1], Flavia Pia Doraci[1], Saskia Kuhl[1], Roland Goldbrunner[1]

[1]University Hospital Cologne, Köln, Deutschland

**Background:**
As receptor tyrosine kinases, the fibroblast growth factor receptor (FGFR) family has various oncogenic effects, such as increased proliferation, invasion or angiogenesis and is thus known to promote metastasis (Korc, M. & Friesel, R. E. 2009). Clinical trials with targeted therapies against FGFR positive tumors have already shown promising outcomes in various cancers. With focus on malignant melanoma, predominantly FGF2 is associated with tumor aggression and progression (Katoh, M. & Nakagama, H. 2014). However, the expression of the FGFR family in brain metastasis of melanoma has not yet been investigated, which therefore was this studies focus.

**Material and methods:**
Brain metastasis of malignant melanoma were collected during neurosurgery. Samples were either grouped according to their BRAF gene mutation status or according to the therapy received by the patients. Transcription rate of FGF1-2 and their receptors FGFR1-4 was quantified via qPCR and normalized with ß-Actin. Statistical analysis was performed with GraphPad Prism.

**Results:**
All FGF genes, except for FRFR-4 were higher transcribed in BRAF mutated tumors. FGFR1 (2.562 vs. 0.869, p=0.0039), FGF1 (1.264 vs. 0.333, p=0.0208) and FGF2 (4.289 vs. 1.609, p=0.0009) appeared with most significance. With a focus on the patient's therapy prior to resection, an increased expression with received therapy was seen for all genes, again except of FGFR4, with significance for FGFR2 (3.923 vs. 0.372, p=0.0107) and FGFR3 (4.302 vs. 0.617, p=0.0130). Therapy that had highest influence varied between the genes.

**Conclusion:**
FGF genes are higher expressed in BRAF mutated tumors and those whose patients received therapy after the primary tumor. FGFR4 showed an opposite expression pattern in all analysis.
